# The Homeodomain Derived Peptide Penetratin Induces Curvature of Fluid Membrane Domains

**DOI:** 10.1371/journal.pone.0001938

**Published:** 2008-04-09

**Authors:** Antonin Lamazière, Claude Wolf, Olivier Lambert, Gérard Chassaing, Germain Trugnan, Jesus Ayala-Sanmartin

**Affiliations:** 1 INSERM, UMR538, CHU Saint Antoine, Paris, France; 2 Université Pierre et Marie Curie, CHU Saint Antoine, Paris, France; 3 UMR 5248 CBMN, CNRS, Université Bordeaux 1, ENITAB, IECB, Pessac, France; 4 UMR CNRS 7613, Université Pierre et Marie Curie, Paris, France; Massachusetts Institute of Technology, United States of America

## Abstract

**Background:**

Protein membrane transduction domains that are able to cross the plasma membrane are present in several transcription factors, such as the homeodomain proteins and the viral proteins such as Tat of HIV-1. Their discovery resulted in both new concepts on the cell communication during development, and the conception of cell penetrating peptide vectors for internalisation of active molecules into cells. A promising cell penetrating peptide is Penetratin, which crosses the cell membranes by a receptor and metabolic energy-independent mechanism. Recent works have claimed that Penetratin and similar peptides are internalized by endocytosis, but other endocytosis-independent mechanisms have been proposed. Endosomes or plasma membranes crossing mechanisms are not well understood. Previously, we have shown that basic peptides induce membrane invaginations suggesting a new mechanism for uptake, “physical endocytosis”.

**Methodology/Principal Findings:**

Herein, we investigate the role of membrane lipid phases on Penetratin induced membrane deformations (liquid ordered such as in “raft” microdomains versus disordered fluid “non-raft” domains) in membrane models. Experimental data show that zwitterionic lipid headgroups take part in the interaction with Penetratin suggesting that the external leaflet lipids of cells plasma membrane are competent for peptide interaction in the absence of net negative charges. NMR and X-ray diffraction data show that the membrane perturbations (tubulation and vesiculation) are associated with an increase in membrane negative curvature. These effects on curvature were observed in the liquid disordered but not in the liquid ordered (raft-like) membrane domains.

**Conclusions/Significance:**

The better understanding of the internalisation mechanisms of protein transduction domains will help both the understanding of the mechanisms of cell communication and the development of potential therapeutic molecular vectors. Here we showed that the membrane targets for these molecules are preferentially the fluid membrane domains and that the mechanism involves the induction of membrane negative curvature. Consequences on cellular uptake are discussed.

## Introduction

The delivery of active molecules into cells requires the step of efficiently cross the plasma membrane barrier. For this purpose, cells have developed messenger proteins containing the so called protein transduction domains (PTD). These domains are usually present in transcription factors, are rich in basic residues and are responsible for the internalisation of the proteins into the cell cytoplasm [1], [2]. This last property resulted not only in new concepts about cell communication, but also in the development of various molecular vectors such as Penetratin, Tat peptide and transportam which comprise the so called cell penetrating peptides (CPP). CPP attached to a pharmacological cargo are judged as potential therapeutic carriers for internalisation of hydrophilic molecules inside eukaryotic cells (for review see [3], [4]). Several CPP have been modelled after particular sequences found in cellular proteins, suggesting that some of them, notably the homeoproteins, could be acting as signals for cellular transduction [1]. Penetratin, a peptide derived from the DNA binding domain of the Antennapedia homeoprotein [5] was extensively studied and was one of the first CPP used to introduce active molecules into cells [6].

Early studies of cell penetration demonstrated that these basic domains cross the membranes independently of receptors and metabolic energy supply [7]. Therefore, the direct interaction with membrane lipids seems to be the clue for their cell uptake. However, more recent works have demonstrated that some peptides are also internalized by endocytosis (for review see [8], [9]). To reconcile the previous observations, several mechanisms for internalisation of basic peptides have been proposed which include different types of endocytosis, “electroporation-like”, inverted micelles, and pores formation [8], [9]. Since Penetratin does not permeabilize membranes, the formation of pores, which is the candidate mechanism for the internalisation of antimicrobial amphipathic peptides has been excluded. Whatever the initial step of cell internalisation involves endocytosis or not, the question of how the peptide crosses the plasma or the endosome membrane to reach the cytoplasm remains unanswered.

In model lipid membranes, it has been established that translocation in large unilamellar vesicles (LUV) is dependent on the membrane surface electrostatic potential which is modulated by the lipid composition [10]. Belrose et al [11] showed that Penetratin was able to change the lamellar propensity in membrane lipids dispersions and prompts for the formation of micelles from phosphatidylcholine (PC)/phosphatidylserine (PS) negatively charged vesicles. In experiments with PC/phosphatidylglycerol (PG) (9/1) Giant Unilamellar Vesicles (GUV), different basic peptides were shown to invaginate membranes in the form of narrow tubular structures [12]–[14]. This metabolic energy-independent “physical endocytosis” may well represent a new mechanism underlying the cellular uptake of basic cell penetrating peptides.

The concept of membrane domains has been developed in the last ten years, and the role of lipid arrangements in cellular membranes functions, organisation, traffic and regulation of signalling pathways have been demonstrated [15], [16]. The role of membrane microdomains in CPP binding and uptake, especially the molecular mechanisms involved are poorly understood. The role of membrane lipid charges in the Penetratin binding has been a subject of controversy. Several studies have suggested that the presence of negatively charged phospholipids is absolutely required for binding [17]–[19], but others have shown that neutral zwitterionic phospholipids are also able to bind the peptide [20], [21]. It has also been shown that cholesterol modulates binding to PC/PG membranes [22], but the role of membrane domains in the cellular uptake and the mechanisms involved were not detailed. The molecular mechanisms involved in the peptide interaction with the membrane phospholipids as well as the role of membrane domains are interesting for vectorisation strategies at both, the peptide (vector) and the lipid membrane (target) levels. Therefore, we have investigated the influence of lipid domain arrangement in regulating the Penetratin-membrane interactions. The influence of the membrane lipid physical state (liquid ordered such as the “raft” microdomains or liquid disordered “non-raft”) on Penetratin induced membrane deformations was studied. Altogether the results indicate that neutral membranes are able to interact with Penetratin which confirms that the phosphate negatively charged groups exposed on the external leaflet of plasma membrane are competent for interaction with the basic peptide. Perturbations of the lamellar arrangement are consistent with alterations of the acyl chain packing. The membrane deformation (tubulation and vesiculation) observed in this study are thus associated with a sharp increase in the negative curvature of the liquid disordered domains which has not been observed in liquid ordered (raft-like) membranes.

## Results

### Penetratin binding to phospholipids

**Figure 1:**
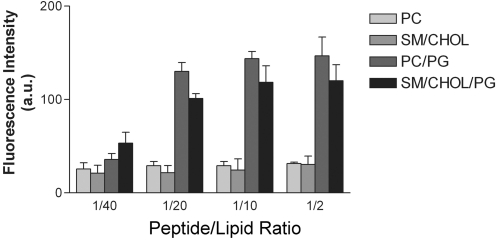
Penetratin binding to LUV. Penetratin binding was followed by detection of tryptophan fluorescence in LUV recovered after centrifugation. Penetratin concentration varied and LUV concentration was maintained constant. Peptide/Lipid ratio is given by weight. In the absence of membranes, no fluorescence was recovered after centrifugation. SM/Chol (1/1), PC/PG (9/1), SM/Chol/PG (4/5/1). (a.u.); arbitrary units. Mean of three independent experiments.

The specificity of membrane phospholipids headgroup in the binding of Penetratin and other basic peptides is debated. Several reports indicate binding to negatively charged phospholipids only [17], [18] but other to zwiterionic phospholipids [20], [21]. The binding of Penetratin to large vesicles (LUV) was quantified monitoring the fluorescence of tryptophan. Figure 1 shows that “neutral” membranes (PC and sphingomyelin (SM)/cholesterol (Chol) (1/1 mol/mol) bind Penetratin at their surface. The presence of 10% negatively charged phospholipids in the membrane bilayer (SM/Chol/PG, 4/5/1 and PC/PG, 9/1) considerably increases the binding of Penetratin as compared with neutral membranes in the absence of negative phospholipids (four fold increase). In the absence of PG, membrane saturation was reached at a peptide/lipid weight ratio of 1/40, but in membranes enriched with 10% PG the plateau of saturation was reached at 1/20. Therefore, negative phospholipids were not required for Penetratin binding but increase considerably its association to membranes.

### Membrane deformations induced by Penetratin on giant unilamellar vesicles (GUV)

**Figure 2:**
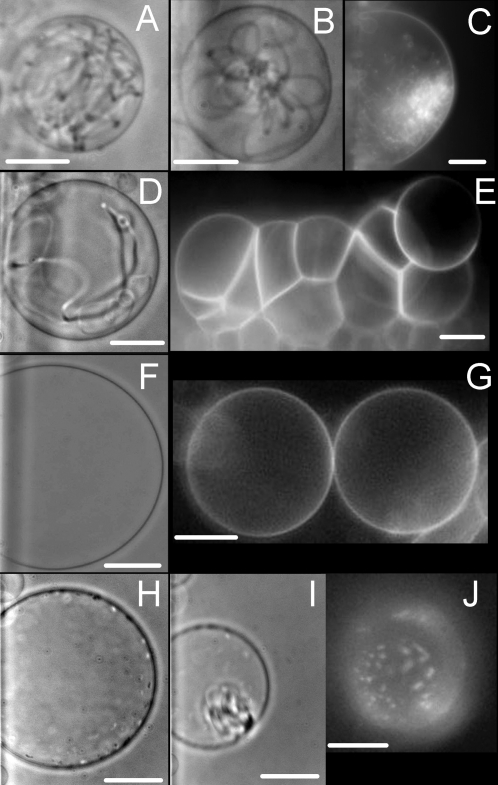
Penetratin effects on GUV in liquid disordered (non-raft) and liquid ordered (raft-like) domains. GUV obtained by electroformation were incubated with Penetratin or fluorescent Penetratin as described in the methods section. Tubulation was only observed in membranes in the liquid disordered state. DOPC (A), egg yolk PC (B,C) and PC/PG (9/1) (D) GUV. Peptide induced vesicles adhesion was observed in all GUV (only shown for PC/PG) (E). Peptides showed no tubulation effect in membranes in the liquid ordered state (“raft-like” SM/Chol (1/1)) (F,G). In SM/Chol membranes supplemented with 10% PG, Penetratin clusterizes at the GUV surface (H,J) and then provokes a grape-like vesiculation phenomenon (I) see also videos S1,S2,S3 in supplement. Dark field images were obtained with CF-Penetratin. Phase contrast images were obtained with cargo-free Penetratin. Bars 20 µm.

In a previous study, we demonstrated that several basic peptides are able to induce the formation of tubular structures and the aggregation of PC/PG (9/1) membranes [14]. Herein, we studied the interaction of Penetratin and Penetratin labelled with the fluorescent cargo 5(6)-Carboxyfluorescein (CF-Penetratin) in membranes mimicking the liquid ordered “raft” microdomains of the plasma membrane (SM/Chol, 1/1) or the liquid disordered “non-raft” fluid domains (PC). We studied also the effect of higher peptide binding by addition of 10% negatively charged phospholipids added to the lipid membranes (SM/Chol/PG, (4/5/1), and PC/PG (9/1)). As shown in figure 2A–D and videos S1 and S2, the fluid disordered membranes exposed to Penetratin form tubular structures. Vesicles adhesion was also detected (Fig 2E and video S1). This is in agreement with a previous study on PC/PG GUV [14], and demonstrates both; the occurrence of tubular structures and vesicle formation in the absence and in the presence of anionic phospholipids, and the capability of Penetratin to form tubular structures and membrane bridges in the presence of a fluorescent cargo. The tubular structures were observed in membranes composed of heterogeneous fatty acid chains PC from biological source (egg yolk), as well as synthetic dioleoylphosphatidylcholine (DOPC) with homogeneous fatty acid chains (Fig 2A). Membrane tubes were observed at temperatures ranging from 10 to 40°C meaning that they are in this range, temperature-independent. In a previous report [14] we observed the formation of tubes of variable diameter obtained with different basic peptides. Herein, we observed that the tubes obtained with Penetratin and CF-Penetratin were thinner in PC GUV compared to those observed in PC/PG GUV (Fig 2A–D). Neither Penetratin nor CF-Penetratin were able to induce tubes in raft-like mimicking GUV (SM/Chol) even at high temperatures (45°C) indicating clearly that the liquid ordered lamellar phase is not susceptible to participate in membrane deformations induced by Penetratin (Fig 2F,G). The absence of effect is not due to the absence of peptide binding as shown by the fluorescent peptide associated to the SM/Chol GUV. However, when these raft-like GUV were supplemented with 10% PG, the peptides induced the formation of peptide aggregates on the GUV surface (dark and fluorescent patches in Fig 2H,J) before the formation of grape-like accumulation of vesicles and GUV collapse (Fig 2I and video S3). In the SM/Chol and SM/Chol/PG GUV, tubes were not formed, but vesicles adhesion was observed at high Penetratin concentrations. Finally, we observed also tubular structures in mixed PC/SM/Chol (1/1/1) GUV in which the liquid ordered and disordered domains coexist (not shown).

### Penetratin induce membrane adhesion and vesicles aggregation

**Figure 3:**
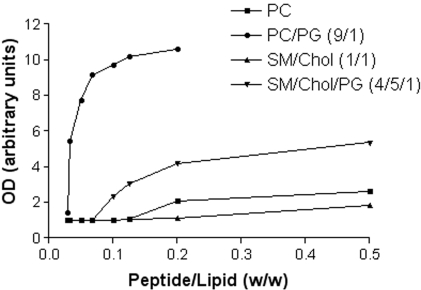
Membrane aggregation by Penetratin. Curves of LUV aggregation as a function of peptide/lipid ratio. LUV aggregation was measured by turbidimetry at plateau (20 minutes after peptide addition). Representative curves of three experiments.

**Figure 4:**
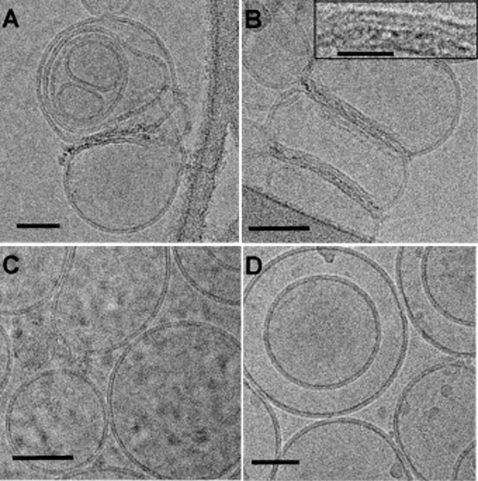
LUV aggregation and deformation by Penetratin as a function of lipid composition. LUV of different composition in the presence of Penetratin were observed by cryo-electron microscopy. PC (A), PC/PG (9/1) (B), SM/Chol (1/1) (C), and SM/Chol/PG (4/5/1) (D). Bars 50 nm. Inset in B shows an amplification of the Penetratin membrane bridges. Notice the perturbation of the phospholipid bilayer. Bar for inset 20 nm.

On the contrary to the membrane tubulation effect which was only observed in GUV formed with the liquid disordered lipid domain, membrane bridging induced by Penetratin was observed in all four types of GUV investigated. The Penetratin capacity to bridge membranes and produce LUV aggregation shows that in the presence of PG, membrane aggregation is clearly more efficient than in it is absence (Fig 3). Comparison of the peptide/lipid ratio to reach the plateau for aggregation of different LUV, PC (P/L 1/5) versus SM/Chol (P/L 1/2) and PC/PG (P/L 1/15) versus SM/Chol/PG (P/L 1/5) shows that membrane aggregation is completed for liquid disordered LUV at lower Penetratin concentration than for liquid ordered LUV. Cryo-electron microscopy observation of aggregated LUV reveal that vesicles in the liquid ordered phase (SM/Chol (1/1) and SM/Chol/PG (4/5/1)) keep a round shape appearance not perturbed by the peptide (Fig 4). On the contrary, the liquid disordered LUV, notably in the presence of PG, have shown membranes deformed by Penetratin interaction, confirming that the fluidity is required to observe the peptide induced deformations and high levels of membrane aggregation. The electron microscopy images reveal a strong perturbation of the membrane bilayer in the peptide-membrane bridges. The measurement of the thickness of one membrane bilayer and the associated Penetratin complex was about 7.00 nm.

### Penetratin induce negative curvature on liquid disordered membranes but not in raft-like membranes

**Figure 5:**
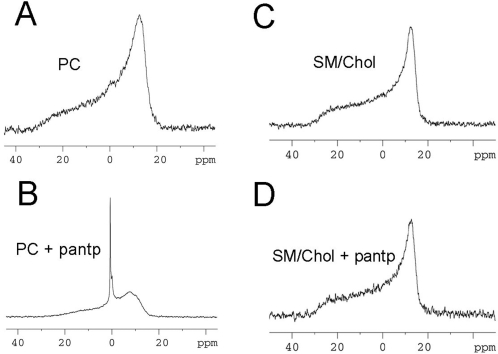
Effect of Penetratin in curvature of multilamellar vesicles. ^31^P-NMR spectra of MLV of different composition in the absence (A,C) and the presence of Penetratin (B,D) at a Peptide/Lipid weight ratio of 1/7. Typical spectra of lamellar phases are observed for both PC and SM/Chol (1/1) preparations in the absence of peptide. In the presence of Penetratin, an isotropic signal was observed for PC membranes consistent with the presence of highly curved membranes (B). Penetratin showed no effect on the liquid ordered (raft-like) membrane (D).

In order to understand the perturbations of membranes induced by Penetratin on a nanoscopic scale we performed ^31^P-NMR and X-ray diffraction experiments. Considering that the presence of 10% PG in the membranes increases only binding but was not required for membrane deformations, we have performed experiments on disordered PC and liquid ordered SM/Chol (1/1) membrane models. These compositions are consistent with the external leaflet of plasma membrane domains in resting cells. ^31^P-NMR spectra were obtained from multilamellar vesicles (MLV) suspension in the absence or in the presence of Penetratin (Peptide/lipid ratio P/L = 1/7). In the absence of Penetratin, the spectra were characteristic of lamellar phases for both lipid compositions (Fig 5A,C). Traces of isotropic phosphorous resonance can only be observed. On the liquid disordered phase (PC), Penetratin induces the appearance of a strong isotropic resonance signal consistent with highly curved phospholipid structures with a rapid reorientation regimen (Fig 5B). On the opposite, the ^31^P-NMR spectra of raft-like liquid ordered phase MLV (SM/Chol (1/1)) do not show change (Fig 5D). The emergence of the isotropic peak was also observed in the presence of Penetratin acting on membranes composed of the ternary mixture PC/SM/Chol (1/1/1) where ordered and disordered domains are known to coexist (data not shown) [23], [24]. The isotropic peaks observed with ^31^P-NMR were consistent with highly curved membranes required for the formation of tubes and vesicles as already observed in GUV.

The ^31^P-NMR spectra suggested that highly curved structures are induced by Penetratin. Therefore, in order to determine whether the lipid lamellar arrangement was maintained, we have recorded small angle X-ray diffraction of fully hydrated MLV. X-ray diffraction is helpful to characterize the lamellar, hexagonal and cubic arrangement of phopholipids. In a previous paper, we showed no effect of Penetratin in PC/PG (9/1) membranes at a low Peptide/lipid ratio of 1/20 [14]. Herein, we have performed X-ray diffraction measurements at a P/L ratio of 1/10.

**Figure 6:**
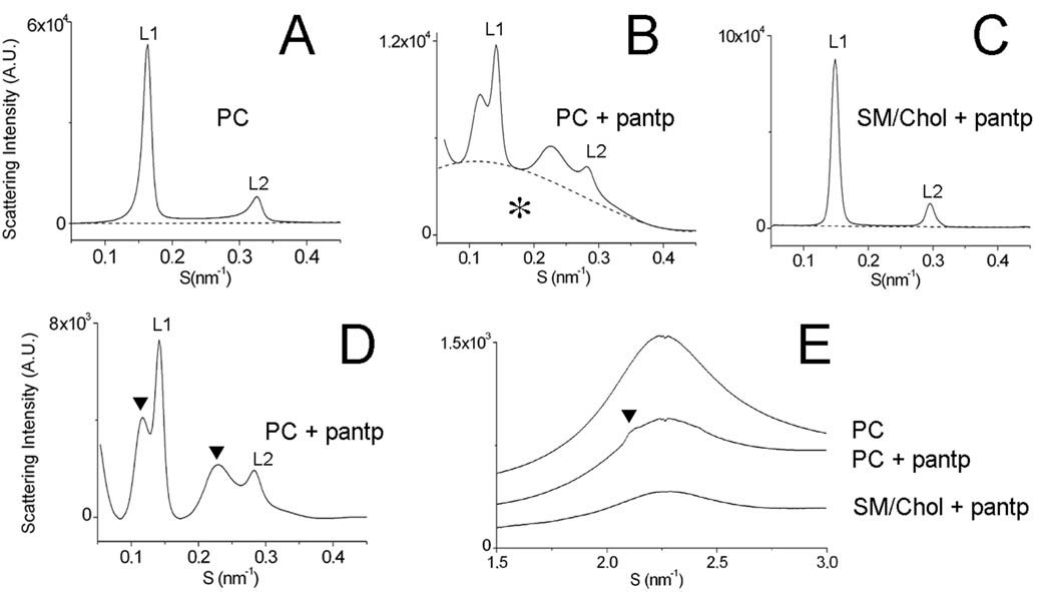
Effects of Penetratin observed by small angle and wide angle X-ray diffraction. A to D small angle X-ray diffraction. Diffractograms are not corrected for the base line; (A) PC membranes, (B) PC membranes in the presence of Penetratin showing a “distorted baseline” (star) corresponding to the form factor contribution. The broad signal was tentatively related to the formation of small vesicles, (C) SM/Chol (1/1) membranes in the presence of Penetratin. (D) Diffractogram is subtracted for the distorted baseline. PC membranes in the presence of Penetratin show first (L1) and second (L2) order Bragg peaks of a lamellar phase (d-spacing in the ratio 1∶1/2). The peaks as compared to (A) in the absence of peptide are shifted to the left indicating an increase in the repeat distance from 6.01 to 7.05 nm. In addition, Penetratin induces the splitting of peaks and the separation of a lamellar phase with considerably larger repeat distance (arrowheads). (E) Effects of Penetratin as observed by the wide angle X-ray diffraction. Peaks of PC MLV membranes are shown in the absence (top) and in the presence of Penetratin (middle). Peak of SM/Chol membranes is also shown in the presence of Penetratin (bottom). The arrowhead indicates the unresolved contribution assumed to correspond to the increased inter-acyl chains spacing. All experiments presented were done at 25°C.

In the absence of peptide, the non-raft PC membranes showed a single lamellar phase (Fig 6A). In the presence of peptide, three different perturbations are observed. Firstly, diffractograms show the disturbance introduced in the presentation of the sample. The regular array of lamellar MLV membranes was altered deeply as revealed by the decrease of the Bragg-peaks intensity and the increase of the form factor contribution of the bilayers (Fig 6B). The alteration is consistent with the vesiculation induced by the peptide. The effect is clearly observed on liquid disordered membrane and it is associated with the decrease in the intensity of the Bragg reflections. The Bragg's peaks are considered to correspond to the constructive interferences between diffracted X-rays by array of regularly spaced lamellae. A second important perturbation of liquid disordered membranes under the influence of Penetratin is the increased repeat distance from 6.01 nm to 7.05 nm for PC in the presence of Penetratin. Thirdly, an additional lamellar structure with a repeat distance of 8.54 nm has appeared in the presence of Penetratin. After baseline correction (Fig 6D), the measurement of spacing suggests that this arrangement is lamellar (2^nd^ order peak spacing close to d/2). Noticeably, under the present conditions, an inverse hexagonal H_II_ geometry which is considered as an intermediate in the formation of inverted micelles was not observed. For the ordered SM/Chol (1/1) membranes, the two peaks of the lamellar phase are clearly visible. Penetratin did not induce the dramatic changes observed with the fluid PC membranes (Fig 6C).

Interestingly, wide angle X-ray diffractograms revealed that the distance of the fatty acyl chains of SM/Chol membranes is not modified by Penetratin. The spacing is maintained at 4.4 Å (Fig 6E). For the liquid disordered phase PC, the spacing of the fatty acyl chains was 4.5 Å. In the presence of Penetratin, an additional peak with a wide spacing of 4.7 Å was detected (Fig 6E). This peak indicates an increase in the fatty acyl chains distances. We assume that the de-packing of acyl chains in the presence of Penetratin is in agreement with two different hypothesis: the induction of negative curvature of phospholipids, and/or the induction of lipid phase separation.

## Discussion

In a previous work, we observed that different basic peptides were able to induce membrane invaginations in giant vesicles, a process suggesting “physical endocytosis” mechanism for the basic peptide uptake [14]. However, the role of lipids in this phenomenon was not studied. In the present paper we investigated the influence of the membrane lipid composition in the changes induced by the homeodomain-derived peptide Penetratin.

Several studies suggest that increase in negatively charged phospholipid concentrations favours Penetratin and other basic peptide sequences binding to membranes by electrostatic adsorption. According to these authors, neutral phospholipids (PC) are unable to bind the peptide or to aggregate membranes [17]–[19], [25]. This discrepancy with our data showing that pure zwitterionic interfaces bind Penetratin can be explained taking into account that in some of the previous studies, the authors used Small Unilamellar Vesicles (SUV) with a highly positive curvature of membranes. We assume that the spreading of neutral phospholipid headgroups could result in the reduction of peptide binding. We have presently used weakly curved LUV and GUV because they have a radius of curvature close to those of cell membranes. In agreement, other studies have demonstrated that basic peptides [26] and Penetratin are able to bind PC monolayers [20] and flat bicelles [21]. Our data indicate that Penetratin binding to membrane bilayers does not depend on the liquid ordered or liquid disordered physical state of the membrane because PC as well as SM/Chol (1/1) vesicles bind Penetratin. The addition of PG (10%) to the membranes increases to four fold the binding. Altogether the data are consistent with the ability of Penetratin to bind lipid membrane possibly by electrostatic interaction of basic residues with anionic headgroups or by forming hydrogen bonds. Guanidinium groups of arginine and the phosphate group of phospholipids are susceptible to act as donor and acceptor in the formation of hydrogen bond at neutral pH [27], [28]. Interestingly, in the case of poly-L-Lysine, a molecule without the guanidinium groups of arginine, the formation of tubes is abolished in GUV lacking anionic phospholipids [12].

Penetratin ability to induce vesicles aggregation by bridging adjacent membranes was correlated with binding [14]. Bridging increases after surface membrane saturation by the charged peptide. Modelisation studies suggest that the amphipathic fusion peptide E5 of *influenza* virus binds strongly and at a deeper distance from the aqueous interface in fluid dimirystoylphosphatidylcholine membranes compared to a rigid dipalmytoylphosphatidylcholine membrane [29]. Here, we observe that membrane fluidity also favours bridging. This may also indicate that mobility of the peptide on the fluid surface favours the arrangement required to form inter-membrane bridges.

Membrane fluidity was critical in the occurrence of membrane deformations after CPP binding. In a membrane comprised of fluid disordered phase (Ld) such as expected for unsaturated PC around physiological temperatures, Penetratin was able to induce invaginations in giant vesicles (PC, DOPC and PC/PG (9/1)). On the contrary, the peptide showed no effect on a raft-like membrane (SM/Chol (1/1)) indicating that the rigidity of the membrane comprised with the ordered phase has restrained considerably tubulation. Roux et al. [30] showed that the force required to form tubes from a liquid ordered membrane is 1.7 times stronger than for a liquid disordered membrane. Penetratin interaction energy is probably not strong enough to deform the ordered rigid domains.

It is noticeable that peptide binding is increased after the addition of PG in the ordered membranes. This induces peptide aggregation and an accumulation of small dimension vesicles (grapes). We have interpreted the occurrence of these grape-like vesicular structures by the peptide inefficiency to support the “normal” elongation of the highly curved thin tubes. Interruption of the tube elongation by membrane rigidity surrounding the tubulation starting point may eventually result in grape vesiculation. The resistance of the raft-like membranes to deformation by the peptide was confirmed by cryo-electron microscopy of LUV in which only the liquid disordered membranes undergo deformations in the peptide-membrane bridges.

The structure of membranes in the presence of Penetratin was also studied by ^31^P-NMR and X-ray diffraction. Both methods revealed that the liquid ordered phases comprised of SM and Chol are resistant to membrane deformation by the peptide. On the contrary, both methods revealed perturbation of the lipid arrangement in the disordered phase. ^31^P-NMR spectra showed a strong isotropic peak consistent with highly curved membrane structures. A possible explanation for the isotropic signal is the fast tumbling of small vesicles detached from the large MLV. Other possible explanations such as the transition of lamellar to cubic arrangement have been turned down by X-ray examination. Vesicle formation is also suggested by the X-ray diffractograms. The X-ray data showed a decrease in the Bragg's reflections corresponding to the lamellar arrangement. Due to vesiculation, the Bragg-peaks diminish and the typical form-factor contribution arising from uncorrelated bilayers becomes visible as the structure factor contribution of constructive interference Bragg's diffraction peaks decreases. It is also shown that the peptide separates partially the phospholipids into two lamellar arrangements with wide distance repeats as regard to the records in the absence of peptide. We hypothesize that the arrangement with the longer spacing corresponds to the increased inter-bilayer distance consistent with the measurement given by cryo-electron microscopy. Indeed, the distance repeat of the lamellar membrane perturbed by Penetratin is 7.05 nm as measured by X-ray diffraction, a value in agreement with the thickness of the lipid bilayer plus the electron dense peptide layer observed by cryo-electron microscopy (∼7 nm). However, the Bragg peaks with the repeat distance of 8.54 nm would represent another lamellar arrangement which is probably related to the vesiculation intermediate arrangement. It might be interesting to speculate whether this thicker structure is related to the rod-like structures observed in membranes incubated with Tat peptide [31]. However, neither a cubic nor an inverted hexagonal phase has been revealed by the present X-ray examination. The enlarged inter-chain distance which was detected by wide angle X-ray diffraction in the presence of Penetratin may be a clue to explain the mechanism of tube or vesicle formation. The enlarged inter-acyl chain distance is in agreement with the hypothesis of a negative curvature induced by the peptide as suggested previously [14], but it might also results from phase separation induced by the formation of peptide-lipid clusters.

In conclusion, we show that the homeodomain-derived basic peptide Penetratin is able to bind several types of membranes (ordered or disordered) but can only induce tubulation (“physical endocytosis”) in liquid disordered membranes. This can be seen in the absence of negatively charged phospholipids. This is relevant to eukaryotic cells where the external layer of the plasma membrane does not contain significant amounts of anionic phospholipids in the resting state. Interaction of Penetratin with the phosphate group of PC or SM of cell plasma membrane would be efficient to ensure the proper binding. Second, the tubulation effect of Penetratin on phospholipid membranes is only possible on membranes in the liquid disordered phase. We assume that this effect is due to the capability of the peptide to induce negative curvature in membranes. No tubule was observed in raft-like liquid ordered membranes exposed to Penetratin. The data suggest that the formation of lipid-peptide complexes, which requires fluidity, is critical and that the compactness of the raft-like domains is a barrier for cell penetration. Fretz has recently shown that perturbation of cell membrane domains by cholesterol depletion with methyl-beta-cyclodextrin increases polyarginine (R8) uptake independently of endocytosis [32] possibly after the transition of the liquid ordered to disordered arrangement. Our data suggest that for the biological processes involving messenger proteins containing protein transduction domains (i.e. TAT and homeoproteins) as well as for therapeutic molecular vectors, the preferential cellular membrane target for penetration would be the non-raft fluid plasma membrane domains. In this case, the formation of invaginations: tubes in liquid disordered domains and vesiculation in mixed ordered/disordered domains could explain the metabolic energy independent mechanism of internalization. Experiments with messenger proteins and peptides on cell membranes are the perspectives for the future research.

## Materials and Methods

### Materials

egg yolk L-α-phosphatidylcholine (PC), egg yolk L-α-phosphatidyl-DL-glycerol (PG), sphingomyeline (SM), cholesterol (Chol), dioleoylphosphatidylcholine (DOPC), and 5(6)-carboxyfluorescein were purchased from Sigma. Penetratin (RQIKIWFQNRRMKWKK) was synthesized and purified as previously described [14]. Resins, Boc and Fmoc-protected amino acids were obtained from Senn Chemicals (Switzerland). Solvents (peptide synthesis grade) and other reagents for peptide synthesis were obtained from Applied Biosystems.

### CF-Penetratin synthesis and purification

The synthesis of carboxyfluoresceinated Penetratin (CF-RQIKIWFQNRRMKWKK) was performed by solid-phase Fastmoc chemistry on an Applied Biosystems 433A automated peptide synthesizer as described in Bruston et al. [33]. Briefly, Synthesis products were cleaved from resin using a solution of trifluoroacetic acid (TFA) (95%) and Triisopropylsilan (2.5%), precipitated in ether, centrifuged and then lyophilized. Peptides were purified by preparative reverse-phase HPLC on a C8 column using a linear acetonitrile gradient in a TFA aqueous solution 0.1% (v/v). Peptides were >95% pure as assessed by analytical HPLC. Peptide identities were checked by MALDI-TOF mass spectrometry (Voyager Elite, PerSeptive Biosystems) using cyano-4-hydroxycinnamic acid matrix.

### Model membranes

Giant unilamellar vesicles (GUV) were obtained by electroformation as described in [14]. Briefly, 1.5 µl of lipids solution was spread on each platinum electrode. The lipid film was dried under a gentle stream of nitrogen. The chamber was placed in an inverted microscope and a thermocouple positioned to monitor the temperature (25°C). The electrodes were then hydrated with 2 ml of a low ionic strength buffer (HEPES 0.5 mM, pH 7.4 and conductivity 20 µS/cm). Immediately after buffer addition, a low frequency alternating field (5 Hz and 1 V) was applied on the electrodes for at least 2 hours. GUV of diameter between 10 to 100 µm were observed. Large unilamellar vesicles (LUV) were prepared by extrusion of MLV through a polycarbonate filter (pore diameter 100 nm) as described in [14]. The ratios of different phospholipids in the membranes are expressed in moles (i.e. SM/Chol 1/1). The Peptide/Lipid ratio is expressed in weight.

### Membrane deformations

The capability of Penetratin to perturb membranes with low curvature was observed by phase contrast microscopy. 2.5 µl of 50 µM Penetratin solution were added close to the electrodes containing the GUV (2 ml of buffer) and images were captured with a CCD camera (Cool SNAP HQ) controlled with Metamorph software (Roper Scientific). Unless specified, the observation temperature was 25°C. The observation of GUV in the presence of CF-Penetratin was performed in the same conditions using a fluorescent illumination (fluo arc N HBO 103, Zeiss) and a Zeiss filter (Ex/Em, 546/590+). Penetratin induced membrane perturbations at nanoscopic scale were observed by cryo-electron microscopy of LUV as described [34]. Briefly, LUV (15 µg) were mixed with 3 µg of Penetratin in buffer HEPES 0.5 mM, pH 7.4. Membrane aggregation was stopped after 15 minutes of incubation. The Penetratin-LUV suspensions were vitrified by plunging in liquid ethane, following standard fast freeze procedures [35]. Sample observations were performed with a Tecnai F20 FEI transmission electron microscope, operating at 200 kV. Low-dose images were recorded at a nominal magnification of 50 000 with a 2k×2k USC1000 slow-scan CCD camera (Gatan, CA, USA).

### Membrane binding and aggregation

Penetratin binding to membranes was assayed by LUV centrifugation at 150 000 g for 30 minutes in a TL100 ultracentrifuge (Beckman) as described in [36]. LUV (0.12 mg ml^−1^) in buffer (HEPES 10 mM, pH 7.4) were incubated with different peptide concentrations for 20 minutes. After centrifugation, the pellet was recovered in the buffer supplemented with 0.05% SDS. Quantification of bound Penetratin in pellets was followed by measuring the intensity of the tryptophan fluorescence maxima. Excitation wavelength was 280 nm, and emission spectrum was collected (290–600 nm).

LUV aggregation was monitored by turbidimetry (absorbance at 340 nm) in a Cary spectrophotometer (Varian) as described [37]. Peptides were added to a 500 µl quartz cuvette containing 10 µg of LUV in a HEPES 10 mM pH 7.4 buffer and the absorbance was followed during 20 min after peptide addition. Membrane aggregation reached the plateau at 20 minutes.

### ^31^P-NMR spectroscopy

All ^31^P-NMR experiments were recorded on a Bruker DMX 500 spectrometer and were processed with the UXNMR software. Measurements were performed at 202.5 MHz, using a single-pulse experiment with a 12.0 µs pulse, 1.2 s relaxation delay time and broadband proton decoupling. Typically, 24 000 scans were acquired. An exponential multiplication, corresponding to a line broadening of 30 Hz, was applied to FID prior to Fourier transformation. Samples were prepared by dissolving dry lipids (20 mg) in chloroform/methanol (2/1, vol/vol) and mixing to obtain the indicated proportions. The solvent was subsequently evaporated under a stream of nitrogen and residual solvent removed under high vacuum for 1 day. In order to obtain the Multiple Lamellar Vesicles (MLV) the dry lipids were hydrated in H_2_O/^2^H_2_O (9/1 vol.) containing 3 mg of Penetratin. All experiments were done at 40°C.

### X-ray diffraction

Small-angle X-ray scattering (SAXS) and wide-angle X-ray scattering (WAXS) measurements were performed at the Synchrotron Radiation Source of Spring8 (Aichi, Japan) following a protocol adapted from Tessier et al. [38] as described in [14]. Briefly, samples were prepared by dissolving dry lipids (20 mg) in chloroform/methanol (2/1, vol/vol) and mixing to obtain the indicated proportions. The solvent was subsequently evaporated under a stream of nitrogen at 45°C and residual solvent removed under high vacuum for 1 day. In order to obtain the Multiple Lamellar Vesicles (MLV) the dry lipids were hydrated with an equal weight of buffer (5 mM HEPES pH 7.4) containing 2 mg of Penetratin. The lipid dispersion was thoroughly stirred, sealed under argon and kept until examination at 4°C. For X-ray measurements, samples (∼20 µl) were deposited between two thin mica windows and mounted on a programmable thermal stage (Linkham). The sample to detector (Rigaku Image Plate) distance is 1 m. Samples were exposed for 30 seconds to the beam. Repeated measurements show reversibility. Spacings were determined from axially integrated 2-D images using the FIT2D program. Silver behenate was used as the distance calibration standard.

## Supporting Information

Video S1 Membrane adhesion by Penetratin. Tube formation and membrane (GUV) adhesion induced by fluorescent Penetratin. Membranes in the liquid disordered phase.
(0.36 MB MPG)

Video S2 Penetratin-induced tubulation. Fluorescent Penetratin forms tubes on liquid disordered membranes (PC/PG (9/1) GUV).
(0.37 MB MPG)

Video S3 Effect of Penetratin on a rigid membrane. Penetratin form patches of aggregated peptide on the GUV surface before grape-like vesiculation. GUV membrane composition (SM/Chol/PG: 4/5/1).
(0.78 MB MPG)
